# Nitrification inhibitors can increase post-harvest nitrous oxide emissions in an intensive vegetable production system

**DOI:** 10.1038/srep43677

**Published:** 2017-03-07

**Authors:** Clemens Scheer, David Rowlings, Mary Firrell, Peter Deuter, Stephen Morris, David Riches, Ian Porter, Peter Grace

**Affiliations:** 1 Institute for Future Environments, Queensland University of Technology, Brisbane, QLD 4000, Australia; 2 Department of Agriculture, Fisheries and Forestry (Queensland), Gatton Research Station, QLD 4343, Australia; 3 NSW Department of Primary Industries, Wollongbar Primary Industries Institute, Wollongbar NSW 2480, Australia; 4 School of Life Sciences, LaTrobe University, Bundoora, Vic 3083, Australia

## Abstract

To investigate the effect of nitrification inhibitors (NIs) 3,4-dimethylpyrazole phosphate (DMPP) and 3-methylpyrazole 1,2,4-triazole (3MP + TZ), on N_2_O emissions and yield from a typical vegetable rotation in sub-tropical Australia we monitored soil N_2_O fluxes continuously over an entire year using an automated greenhouse gas measurement system. The temporal variation of N_2_O fluxes showed only low emissions over the vegetable cropping phases, but significantly higher emissions were observed post-harvest accounting for 50–70% of the annual emissions. NIs reduced N_2_O emissions by 20–60% over the vegetable cropping phases; however, this mitigation was offset by elevated N_2_O emissions from the NIs treatments over the post-harvest fallow period. Annual N_2_O emissions from the conventional fertiliser, the DMPP treatment, and the 3MP + TZ treatment were 1.3, 1.1 and 1.6 (sem = 0.2) kg-N ha^−1^ year^−1^, respectively. This study highlights that the use of NIs in vegetable systems can lead to elevated N_2_O emissions by storing N in the soil profile that is available to soil microbes during the decomposition of the vegetable residues. Hence the use of NIs in vegetable systems has to be treated carefully and fertiliser rates need to be adjusted to avoid an oversupply of N during the post-harvest phase.

Agricultural activities are responsible for about 70% of anthropogenic nitrous oxide (N_2_O) emissions, a potent greenhouse gas with a global warming potential nearly 300 times that of CO_2_ and arguably the most important form of environmental nitrogen (N) pollution^1^. Use of N fertiliser and animal manure are the main sources of atmospheric N_2_O, and N_2_O emissions are predicted to double by 2050^1^,^2^. Vegetable cropping systems cover approximately 7% of the global agricultural area and are characterized by high N application rates, frequent irrigation and several tillage–planting cycles per year^3^. In addition to the high fertiliser N inputs vegetable crop residues typically have a low C/N ratio (8 to 17) and large amounts of N are incorporated into the soil after harvest (up to 450 kg N ha^−1^ yr^−1^)^4^. Such residues are decomposed rapidly and release mineral N and readily available C into the soil, which combined with high O_2_ consumption rates during residue decomposition, can create anaerobic microsites in the soil and in turn enhance denitrification resulting in high long, lasting fluxes of CO_2_ and N_2_O following incorporation^5^. As a consequence these systems are highly susceptible to N losses, thus environmental N pollution from intensively cropped vegetable fields has been of great concern in recent years. Extraordinarily high emissions of N_2_O (up to 240 kg N_2_O-N ha^−1^yr^−1^) have been reported from heavily fertilised sub-tropical vegetable production systems in China^6^, and it is estimated that globally 45 Mt CO_2_−eq. per year are emitted from synthetic fertiliser used in vegetable production systems^3^. However, the majority of published studies come from vegetable systems in China and extrapolate total annual output from relatively short duration monitoring so the reproducibility of these estimates is untested.

It has been shown that ammonia oxidation dominates N_2_O production in NH_4_^+^-fertilized vegetable soils^7^. Therefore, the use of nitrification inhibitors (NIs) could be an effective option to increase N fertiliser use efficiency and decrease N_2_O emissions in vegetable cropping systems^8^. NIs inhibit soil microbial nitrification resulting in lower soil NO_3_^−^ levels and reduced denitrification and N_2_O production. Different studies have shown that NIs can reduce N_2_O emissions from cropping soils^9^,^10^. A recent meta-analysis suggests that the NI 3,4-dimethylpyrazole phosphate (DMPP) decreases N_2_O emissions and total N losses in cropping systems by 48% and 27%, respectively^11^. However, most data on the effect of NIs on N_2_O emissions and productivity refer to forage or cereal systems or laboratory experiments and the efficacy of many NIs in vegetable production systems remains unknown. Such data is urgently needed to identify management strategies that maximise the efficient use of fertiliser N while minimising environmental impacts from intensive vegetable production.

We conducted a field experiment to investigate the effect of two NIs on N_2_O emissions, soil inorganic N and yield in a sub-tropical vegetable production system in Australia over 355 days using a high frequency automated greenhouse gas measurement system. The two main objectives of the study were: to investigate N_2_O emissions (including EFs) and vegetable yield from a typical vegetable rotation in response to different N fertiliser products; and to test the assumption that the use of NI will decrease N_2_O emissions.

## Results

### Seasonal variability of environmental and soil conditions

**Figure 1:**
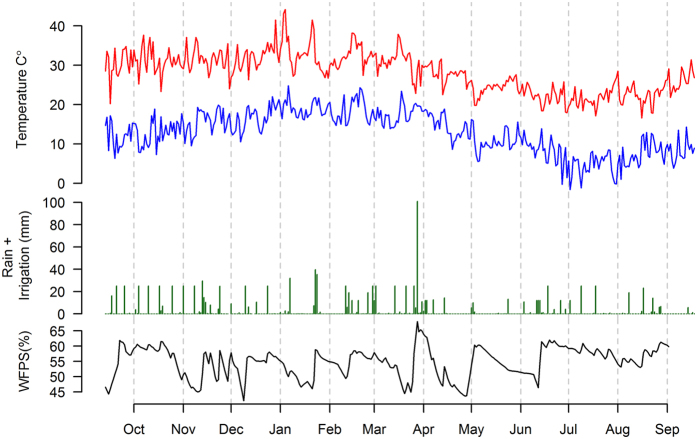
Maximum (red line) and minimum (blue line) hourly air temperature, daily precipitation or irrigation and water filled pore space (WFPS) (0–10 cm) over the one year observation period at the Gatton Research Facility, Australia.

Over the 1 year observation period 587 mm of rainfall was recorded at the study site. In addition the site received 645 mm of irrigation amounting to a total of 1231 mm of total water input. This rainfall was 25% lower that the long term annual precipitation (770 mm). The mean air temperature during the study period was 20.8 °C; maximum hourly air temperature (44.2 °C) was recorded in January 2013, while minimum hourly air temperature (1.6 °C) was recorded in July 2014 (Fig. 1).

#### N_2_O emissions and emission factors

**Figure 2:**
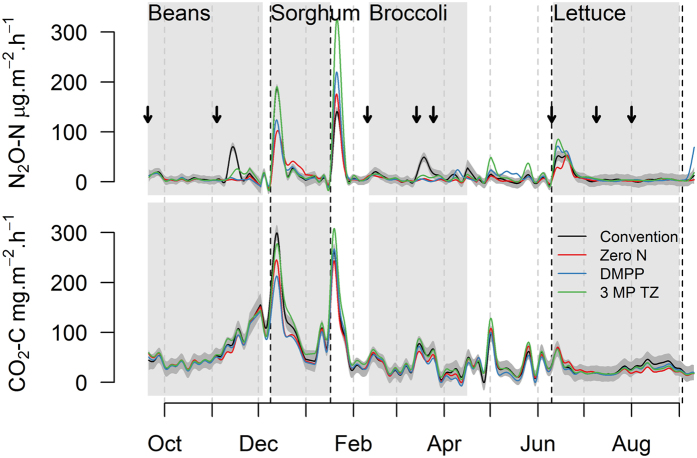
N_2_O and CO_2_ fluxes for the different fertilizer treatments at the Research Facility. Grey polygon encloses approximate 95% confidence limits for conventional N treatment. Arrows indicate the time of fertilizer application. Dashed lines indicate incorporation of crop residues.

The majority of N_2_O fluxes occurred in response to fertilisation events and post-harvest after the incorporation of vegetable residues into the soil (Fig. 2). The first N_2_O emission peak was observed in early November 2013 after the first fertiliser side dressing (36 kg-N ha^−1^ Urea) with significantly elevated emissions in the CONV treatment. Overall highest emissions were observed after the incorporation of vegetable residues into the soil in December and January. The incorporation of the green bean residues on December 9, 2013 resulted in approx. 2 weeks of elevated N_2_O emissions reaching peak emissions of 30–50 g N_2_O-N ha^−1^day^−1^ in the different treatments with significantly lower emissions in the DMPP and 0 N treatment. The incorporation of the forage sorghum on January 17, 2014 resulted in an even higher N_2_O emission pulse reaching peak emissions of 40–80 g N_2_O-N ha^−1^day^−1^ in the different treatments with significantly elevated emissions in the DMPP and 3MP + TZ treatment (Fig. 2). The fertiliser side dressing over the broccoli cropping period resulted in small emission pulses with highest emission in the CONV treatment. After harvest of the broccoli the residues were not incorporated directly but mulched and left on the soil surface. The mulched residues were then incorporated 10 days later, the same day as bed forming and basal fertilisation for the lettuce planting took place. This caused a series of small emission pulses with significantly higher emissions observed in the inhibitor treatments (DMPP and 3MP + TZ).

Cumulative N_2_O emissions were estimated to be 1.11, 1.56, and 1.27 kg N_2_O-N ha^−1^ yr^−1^ for the DMPP, 3MP + TZ and CONV treatments, respectively, while from the unfertilized plots an average of 0.85 kg N_2_O-N ha^−1^ yr^−1^ was emitted (Table 1). The use of DMPP reduced annual emissions by 13%, while the use of 3MP + TZ increased annual emission by 23% compared to the CONV treatment. However, overall there was no significant treatment effect on annual N_2_O emissions due to a high spatial variation within the single replicates.

Corresponding N fertilizer-induced annual N_2_O emission factors (EF’s) found in the present study were 0.08%, 0.21% and 0.13% for the for DMPP, 3MP + TZ and CONV treatments, respectively (Table 1).

#### CO_2_ emissions

CO_2_ fluxes followed a similar pattern as N_2_O showing a response to rainfall and irrigation events with highest emissions post-harvest after the incorporation of vegetable residues into the soil in December and January (Fig. 2). The incorporation of the green bean residues caused elevated CO_2_ emissions reaching peak emissions of 450–570 mg CO_2_-C m^−2^ h^−1^ in the different treatments with lowest emissions in the DMPP and highest in the CONV treatment. The incorporation of the forage sorghum resulted in a similar CO_2_ emission pulse reaching peak emissions of 400–500 mg CO_2_-C m^−2^ h^−1^ in the different treatments with significantly elevated emissions in the 3MP + TZ treatment. Cumulative annual CO_2_ emissions were estimated to be 14.8, 18.5, and 16.3 Mg CO_2_ ha^−1^ yr^−1^ for the DMPP, 3MP + TZ and CONV treatments, respectively, while from the unfertilized plots an average of 15.6 Mg CO_2_ ha^−1^ yr^−1^ was emitted (Table 1). The use of DMPP reduced CO_2_ emissions by 9%, while the use of 3MP + TZ increased annual emission by 14% compared to the CONV treatment with no significant treatment effect on cumulative annual CO_2_ emissions.

#### Vegetable Yield

Total green bean yield ranged from 8.7 to 11.7 (se 1.1) Mg ha^−1^, total broccoli yield ranged from 4.4 to 9.7 (se 0.7) Mg ha^−1^ and total lettuce yield from 41.0 to 68.0 (se 3.9) Mg ha^−1^ in the different fertiliser treatments (Table 2). There was a significant effect of fertiliser application on broccoli and lettuce yield where total yield increased by almost 100% with the application of fertiliser, but we did not find any significant effect of fertiliser application on the green bean yield. There was also no significant difference in yield between the treatments with nitrification inhibitor (DMPP, 3MP + TZ) and the standard fertiliser treatment (CONV).

### Soil mineral N

**Figure 3:**
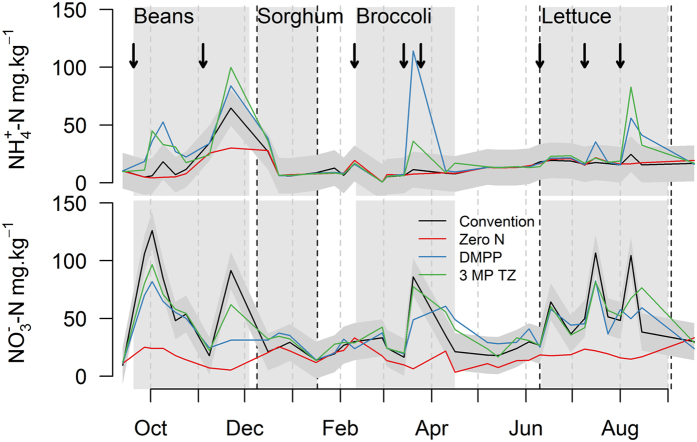
Soil ammonium and soil nitrate content (0–10 cm) over the study period at the Gatton research station, Queensland. Grey polygon encloses approximate 95% confidence limits for conventional N treatment. Arrows indicate the time of fertilizer application. Dashed lines indicate incorporation of crop residues.

Soil NH_4_^+^ and NO_3_^−^ concentrations in the surface soils varied in response to fertilisation events and there was a significant effect of the NI treatments (Fig. 3). Over the year NH_4_^+^ concentrations ranged from 0.6 to 114.2 mg-N kg^−1^ soil dry weight in the different treatments. The application of NIs increased soil NH_4_^+^ concentrations significantly while there was no difference between the CONV and the Zero N treatment. Average soil NH_4_^+^ concentrations over the year ranged from 13.0 ± 0.3, 14.7 ± 0.4, 23.9 ± 2.2 and 21.7 ± 11.9 mg-N kg^−1^ in the Zero N, CONV, DMPP and 3MP + TZ treatments respectively.

Soil 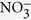 concentrations varied from 3.4 to 126.2 mg-N kg^−1^ in the different treatments (Fig. 3) with average values over the entire experiment ranging from 17.3 ± 1.7, 44.8 ± 2.9, 41.0 ± 1.8 and 44.2 ± 0.8 mg-N kg^−1^ in the Zero N, CONV, DMPP and 3MP + TZ treatments, respectively. There was no significant effect of the NIs on average NO_3_^−^ concentrations compared to the CONV treatments, but NO_3_^−^ concentrations were significantly reduced by the NIs following fertiliser applications in the green beans and lettuce cropping period (Fig. 3).

## Discussion

This is the first study to report on the effect of NIs on N_2_O and CO_2_ emissions from an intensive vegetable crop rotation based on automated high frequency flux measurements. High frequency measurements are necessary to overcome the current uncertainty of N_2_O estimates from vegetable systems and to accurately quantify the inhibition effects of NIs on N_2_O emissions^12^,^13^. Overall N_2_O losses were low compared to emissions found in other intensive vegetable systems, where often extremely high N_2_O emissions (in excess of 20 kg N_2_O-N ha^−1^ yr^−1^) have been reported^6^,^14^,^15^. The substantially lower N_2_O emissions can mainly be attributed to the lower fertiliser N application rates used in our study. Our system received a fertiliser rate of 310 kg-N ha^−1^, while many intensively managed vegetable systems are characterised by extremely high N inputs, often exceeding 1000 kg-N ha^−1^ yr^−1^ 6,14,15. In particular the combination of high mineral N fertiliser rates with high levels of manure application has been shown to stimulate N_2_O losses from denitrification in vegetable systems^16^. However, a study by De Rosa, *et al*.^17^ found similarly low emissions (0.95 to 1.75 kg N_2_O-N ha^−1^ yr^−1^) from a vegetable rotation at the same experimental site as in this study using a combination of manure and mineral fertiliser (up to 500 kg N ha^−1^ yr^−1^ applied). This shows that the alluvial soils in this vegetable production region have only a limited N_2_O emission potential, which is further supported by comparably low N_2_O emissions reported from experimental sites with differing soil texture in the same region^5^,^18^. The reasons for these substantial differences in N_2_O emission potential from different vegetable systems are not entirely clear. Environmental conditions and management in our study are typically attributed to high N_2_O emissions: high rainfall/irrigation and soil temperatures in the sub-tropical climate, high N fertiliser rates (310 kg N ha^−1^ yr^−1^), incorporation of vegetable residues and frequent tillage. Therefore, we presume that the comparable low N_2_O losses observed are mainly related to the soil characteristics at our study site. Soil pH is known to be a principal control of soil denitrification and a positive relationship between soil pH and the N_2_/N_2_O product ratio has often been reported^19^. Therefore the soil pH of 8.2 at our site may have resulted in high N_2_/N_2_O product ratio and the low SOC content (1.5%) limited denitrifier activity, thus explaining the comparably low N_2_O emissions in this sub-tropical vegetable production system.

Annual emission factors, corrected for background emissions, ranged from 0.08 to 0.21% of total fertiliser N applied for the different treatments. These are in line with the results of De Rosa, *et al*.^17^ showing that EFs in this vegetable production region are considerably lower than the IPCC default value (1% of N applied)^20^. They are also at the lower end of EFs reported for other intensively managed vegetable systems. In a recent meta-analysis Rashti, *et al*.^3^ reported seasonal EFs from vegetable production ranging from 0.07 to 5.11% with a global average of 0.94%. This high uncertainty of EFs emphasizes the need to better understand what drives N_2_O emissions in vegetable production systems and demonstrates that regional EFs are better suited to reliably estimate N_2_O emissions from vegetable crops. It is also noteworthy that the EF presented in this study accounts for direct fertiliser induced emissions only and does not consider emissions stemming from N returned to the soil with the vegetable residues. The IPCC methodology assumes that emissions from unfertilised soils are the equivalent of background emissions; however, it has been acknowledged that emissions from unfertilised soils may be greater than “natural emissions” due to mineral N released from the mineralisation of plant residues and/or soil organic matter^21^. This is of particular importance in a system where 50 to 70% of the total emissions occurred after incorporation of crop residues during the post-harvest phase in both the fertilised and Zero N treatments. This should be taken into account for N_2_O emission inventories of vegetable cropping systems.

The temporal variation of N_2_O fluxes showed only low emissions over the vegetable cropping phases, but significantly elevated emissions were observed post-harvest following vegetable residue incorporation into the soil. Overall 50 to 70% of the total emissions occurred during the post-harvest phase (Fig. 2). These results are in line with previous studies from vegetable systems in Australia^5^, and Europe^22^,^23^, which have shown that the incorporation of the vegetable residues can lead to relatively high and long lasting post-harvest N_2_O fluxes. These emission pulses are caused by the rapid decomposition of the vegetable residues as evidenced by the high soil CO_2_ emissions after residue incorporation. This rapid decomposition of crop residues can create anaerobic microsites in the soil which combined with an increased NO_3_^−^ supply from residual N in the soil result in highly elevated denitrification rates and show that crop residue management is an important factor influencing soil N transformations and N_2_O emissions in vegetable production systems.

There was a clear effect of the addition of NIs on N_2_O emissions and soil mineral N content over the beans and broccoli cropping phase where the application of NI reduced N_2_O emissions by 20–60% compared to the standard practice, with DMPP showing a greater mitigation potential. This demonstrates that the NIs were effective in reducing nitrification and subsequent denitrification and consequently decreased soil N_2_O production. This effect is further corroborated by the increased soil NH_4_^+^ concentrations in the NIs treatments, and the increased NO_3_^−^ concentrations in the CONV treatment (Fig. 3). This is in agreement with the reductive effect of NI reported from cereal cropping systems under similar soil and climatic conditions, where a N_2_O emission reduction ranging from 30–80% has been reported^24^,^25^,^26^. It is also within the range of reduction reported for intensively managed vegetable systems^5^,^15^,^16^,^23^,^27^,^28^. However, it needs to be noted that only a few studies have reported on the effect of NIs on N_2_O fluxes in vegetable systems and only three studies have covered an experimental period of at least one year accounting for repeated fertiliser applications and post-harvest effects^15^,^23^,^29^. In our study N_2_O mitigation over the vegetable cropping period was offset by periods of elevated N_2_O emissions from the NI treatments in the post-harvest period of sorghum and broccoli (Fig. 2). This can most likely be attributed to elevated levels of N in the NI treatments during the post-harvest phase and demonstrates that the NIs were effective in storing N in the soil profile. This N was available to soil microbes during the decomposition of the vegetable residues over the post-harvest phase resulting in elevated N_2_O and CO_2_ emissions. Consequently, there was no significant effect of the NIs on cumulative N_2_O emissions and the use of 3MP + TZ even increased annual emission by 23% compared to the CONV treatment.

Similar to N_2_O, there was no significant effect of the NIs on total CO_2_ emissions over one year. However, NIs had an effect on CO_2_ fluxes after residue incorporation where emissions were either reduced or increased in the NI treatments resulting in slightly increased (14%) or decreased (10%) cumulative CO_2_ fluxes in the DMPP and 3MP + TZ treatment, respectively. The effect of NIs on soil C mineralization, as quantified by CO_2_ emissions is still unclear and needs further investigations. While some studies reported a decreased CO_2_ release following the application of NIs^23^,^30^, others observed no effect^5^,^31^. Since there was no effect of the NIs on CO_2_ emissions after their application during the crop growth phase there is no indication of a direct effect of the NIs on soil microbial activity. However, NIs appeared to indirectly influence CO_2_ emissions by affecting the availability of N to soil microbes and therefore residue decomposition.

There was no significant effect of N fertilizer addition on the green bean yield, which can most likely be attributed to the fact that green beans are a legume and therefore capable of fixing nitrogen under N limited conditions. There was also no significant effect of the NIs on crop yield compared to the conventional treatment. This is in contrast to other studies where it has been shown that N fertilizers combined with NIs, such as DCD or DMPP, may improve the yield and quality of vegetable crops^15^,^29^,^32^. However, data on the effect of NI on crop yield is not consistent and it is known that the effectiveness of NIs strongly depends on environmental and management factors^9^. We presume that in our study, the conventional fertiliser dose was sufficient to achieve the yield potential and hence any reduction in N losses via leaching or denitrification did not result in a yield increase. It is also not clear how the effect of NIs on crop yield depends on the N-source preference of the different crops. Plants use organic N, NH_4_^+^, and NO_3_^−^ as N sources, but often cannot successfully compete with soil microorganisms for organic N and NH_4_^+^ 31. In addition, NH_4_^+^ can be immobilised by adsorption onto the cation exchange sites of clay soils and is also used by soil microorganisms as an energy source via nitrification. For that reason, NO_3_^−^ is the major N source for most plants^33^; although the N-source preference of many vegetable crops is still unknown. As a consequence, the use of NI could result in increased NH_4_^+^ immobilisation and reduced NO_3_^−^ supply which could potentially constrain plant N uptake, particularly in these heavy clay soils. However, the overall effect of the NIs on soil NH_4_^+^ and NO_3_^−^ levels and the efficacy in reducing N_2_O over the cropping season in this study indicate that the use of NIs combined with modified fertiliser application rates could be a feasible strategy to reduce N_2_O emissions whilst sustaining high yields. More research is required to optimise N rates and timing for different NIs products and how these can be used to reduce N losses over the post-harvest fallow phase.

This study highlights that N_2_O emissions from irrigated vegetable systems on a black Vertisol in sub-tropical Australia are comparably low and that the proportion of fertiliser N lost as N_2_O from such a system is likely to be considerably lower than the IPCC default value. This demonstrates the need to use the local emission factors to reliably estimate N_2_O emissions from vegetable cropping systems in Australia. It also demonstrates that the use of NIs can lead to elevated N_2_O emissions by storing N in the soil profile that is available to soil microbes during the decomposition of the vegetable residues over the post-harvest phase. Hence the use of NIs in vegetable systems has to be treated carefully and fertilizer rates need to be adjusted to avoid excess soil nitrogen during the postharvest phase. Further research is required to evaluate if the use of NIs in vegetable cropping systems allows for a reduction of the N fertiliser rate. Such a reduction would be necessary to compensate for the increased costs of NI fertiliser and could potentially avoid elevated N_2_O emissions post-harvest.

## Materials and Methods

### Site description

The study was conducted from September 2013 to September 2014 an the Gatton Research Facility, located in the Lockyer Valley approximately 80 km west of Brisbane, Queensland, Australia (latitude 27°33′S, longitude 152°20′E, 94 m above sea level). The Lockyer Valley is a major vegetable producing region in south-east Queensland, characterized by a humid subtropical climate with a humid summer and mild to cool winters. The mean daily minimum and maximum temperatures are 18.7 and 31.2 °C in the summer, and 6.8 and 21.4 °C in winter. Summer rainfall accounts for nearly 60% of the long term average annual precipitation of 770 mm (Bureau of Meteorology, Australia). The soil was formed in an alluvial fan of basalt rock and is classified as a Black Vertisol^34^ which is characterized by high shrink-swell potential due to its high montmorillonite clay content (>50%). Soil chemical and physical characteristics are given in Table 3.

#### Field management and experimental design

A field experiment was conducted over a typical south-east Queensland vegetable crop rotation comprising a succession of three vegetable crops plus a catch crop; green beans (*Phaseolus vulgaris*), broccoli (*Brassica oleracea*) and lettuce (*Lactuca sativa*). Forage sorghum (*Sorghum bicolor*) was used as a catch crop to reduce N losses during the fallow period between the green bean and broccoli crop. A time line of the experiment is shown in Table 4.

The experiment was conducted using four fertiliser treatments with three replications arranged in a randomized complete block design. Each experimental plot was 1.5 m wide × 10 m in length. Fertilized plots received a total of 310 kg-N ha^−1^yr^−1^. This was based on a best practice standard rate of N application for each vegetable crop in the Lockyer Valley. Fertilizer was applied by hand ensuring even application over each plot and incorporated using a rotary hoe just prior to planting seed or transplanting seedlings. Vegetable crops were established using standard commercial practice. Green Beans (cv. Venice) were planted from seed, with 2 rows and 267 plants per plot. Rows were 75 cm apart and plants 7.5 cm apart in the row; Broccoli (cv. Aurora) were transplanted using container grown seedlings, with 2 rows and 67 plants per plot. Rows were 55 cm apart and plants 30 cm apart in the row; Lettuce (cv. Icehouse 2) were transplanted from container grown seedlings, with 3 rows and 86 plants per plot. Rows were 35 cm apart and plants 35 cm apart in the row. A 1 m wide buffer zone was included between plots.

The plots were irrigated with bore water using a hand-shift sprinkler irrigation system. Irrigation was applied during times with low wind speeds to assure uniformity of application. Irrigation amounts were measured using a rain gauge installed at the centre of the experimental plot and were scheduled on a weekly basis, except when sufficient rain had fallen in the previous week.

The fertilizer treatments were:

(i) ZERO N – i.e. no added fertilizer.

(ii) STANDARD GROWER PRACTICE (CONV) – i.e. Nitrophoska^®^ and urea fertilizer application following standard grower practice.

(iii) DMPP – addition of the nitrification inhibitor 3,4-dimethyl-pyrazole phosphate (DMPP), trade name ENTEC^®^ to Nitrophoska and urea, basal and side dressing fertilizer, respectively.

(iv) 3MP + TZ – addition of the nitrification inhibitor combination of 3-methylpyrazole (3-MP) and 1,2,4-triazole (TZ), trade name PIADIN^®^ to Nitrophoska and urea, basal and side dressing fertilizer, respectively.

#### Harvest and Yield Data Collection

Each of the three vegetable crops was harvested when commercially mature. Multiple harvests were conducted for broccoli and lettuce and a once over harvest was conducted for green beans, as per commercial practice in the Lockyer Valley. Maturity was determined using colour, shape, size and defects descriptions listed in commercially available product specifications. The harvest date was determined by regular field inspections in the 10 days leading up to the expected harvest date. Actual harvest dates are listed in Table 4.

#### Measurement of N_2_O emissions

N_2_O fluxes were measured from every plot over one year using a mobile fully automated measuring system similar to the one described in Scheer, *et al*.^5^.

Briefly, the system consisted of 12 acrylic static chambers (50 cm × 50 cm × 15 cm) equipped with pneumatically operated lids and fixed on stainless steel bases inserted 10 cm into the soil. The chambers were linked to a fully-automated system comprised of a computerised sampling unit and an *in situ* gas chromatograph (SRI GC 8610 C) equipped with a ^63^Ni electron capture detector (ECD) for N_2_O concentration analysis. Sample gas measurements were calibrated automatically by a single-point calibration using a certified gas standard of 0.5 ppm N_2_O. During the measuring season a multi-point calibration was performed using certified gas standards of 500, 980, 5030 ppb N_2_O (BOC; Munich, Germany) and the GC response over this range was determined to be linear. The detection limit of the system was ~1.0 μg N_2_O-N m^–2^ h^–1^ and sample dilution via leakage was considered negligible.

#### Flux calculations

Fluxes of N_2_O from the automated chambers were calculated from the estimated rate of increase in concentration over four time points measured during chamber closure as described in detail in Scheer, *et al*.^5^. The coefficient of determination (*r*^2^) was used to quality check the flux measurements. Fluxes were discarded if the regression coefficient of determination (*r*^2^) was <0.80.

Emission factors of the N fertiliser applied to the soil were calculated using the following equation:


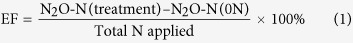


where EF is the emission factor (percentage of the total fertiliser N applied that was emitted as N_2_O-N); N_2_O-N is the total N_2_O over one year (kg N ha^−1^ year^−1^) for each treatment; total N applied is the amount of N fertiliser applied (kg N ha^−1^ year^−1^).

#### Auxiliary measurements

High resolution soil temperature (at a depth of 10 cm) and chamber air temperature was measured every minute in conjunction with the automatic sampling system using a PT100 probe (Temperature Controls Pty, Australia). Soil moisture was measured from 0–10 cm continuously in one plot per treatment using a MP406 standing wave soil moisture probe (ICT International Pty Ltd, Armidale, NSW, Australia) that was calibrated for the soil at the research site. Water-filled pore space (WFPS) was calculated using measured soil bulk density data (arithmetic means of four samples) and an assumed particle density of 2.65 g cm^−1^. The mineral N content of the surface soil (0–10 cm) was measured immediately before planting and weekly to fortnightly over the growing season and the fallow period. At each sampling date, eight samples were taken randomly from each replicate plot with a soil auger and combined to a bulk sample, resulting in three replicate samples per treatment. NO_3_^−^ and NH_4_^+^ were extracted from the soil samples by adding 80 ml of 2 M KCl to 20 g of field-moist soil and shaking it for 1 h. Concentrations of NO_3_^−^ and NH_4_^+^ in the extracts were measured calorimetrically using a discrete analyser (SEAL AQ2+, SEAL Analytical Inc., USA).

#### Statistical Analysis

Linear mixed models were employed to estimate and compare trends in gas and soil nitrogen due to fertiliser regime. The models defined a linear response over time allowed to vary with treatment. The models included a cubic spline basis to allow for curvature about the linear response and to smooth through high frequency variation. A term allowing the cubic spline to depend on treatment was also included. Random effects defining a trend for each plot allowed for intra plot correlation between the repeated observations. Null hypothesis significance tests were assessed by Wald statistics. Estimated trends over time for each fertiliser regime were predicted and presented graphically with +/− twice the standard error of predictions used to approximate a 95% confidence interval.

Cumulative gas emissions from each plot were approximated by integration of flux estimates over time using the trapezoidal rule. Effects of treatment on total emissions were assessed by two-way analysis of variance which estimated variability in total flux associated with experimental block and treatments. The null hypothesis significance test for treatment was conducted by F-ratio test.

## Additional Information

**How to cite this article:** Scheer, C. *et al*. Nitrification inhibitors can increase post-harvest nitrous oxide emissions in an intensive vegetable production system. *Sci. Rep.* **7**, 43677; doi: 10.1038/srep43677 (2017).

**Publisher's note:** Springer Nature remains neutral with regard to jurisdictional claims in published maps and institutional affiliations.

## Figures and Tables

**Table 1:** Average cumulative CO_2_ [Mg CO_2_ ha^−1^ year^−1^] and N_2_O [kg-N ha^−1^ year^−1^] fluxes with corresponding N_2_O emission factors from the different fertiliser treatments with standard error of the means (se).

	Annual CO_2_ Flux [Mg CO_2_ ha^−1^ year^−1^]	Annual N_2_O Flux [kg-N ha^−1^ year^−1^]	Emission Factor [%]
Zero N	14.8^a^	0.85^a^	—
DMPP	15.6^a^	1.11^a^	0.08
3MP + TZ	18.5^a^	1.56^a^	0.21
CONV	16.3^a^	1.27^a^	0.13
se	1.98	0.17	0.04

Means denoted by a different letter indicate significant differences between the treatments (p < 0.05).

**Table 2:** Total yield [Mg ha^−1^] for the three vegetable crops with standard error of the means (se) and least significant difference at 5% critical value.

Treatment	Green beans	Broccoli	Lettuce
CONV	8.7^a^	9.7^a^	68.0^a^
DMPP	11.7^a^	9.1^a^	65.9^a^
3MP + TZ	8.9^a^	8.2^a^	62.5^a^
Zero N	8.9^a^	4.4^b^	41.0^b^
SE	1.1	0.7	3.9
LSD	4.9	2.5	15.5

**Table 3:** Soil chemical and physical characteristic of the experimental site - Gatton Research Facility, Gatton, Queensland, Australia.

Soil property	0–10 cm
Organic Carbon (%)	1.45
Total N (%)	0.11
pH (H_2_O)	8.2
CEC (meq/100 g)	41.0
Clay (%)	60
Silt (%)	22
Sand (%)	18

**Table 4:** Crop management during the experiment.

Date	Management
**Green beans**	
20 Sept. 2013	Transplanting of seedlings
20 Sept. 2013	Basal fertiliser application (54 kg-N ha^−1^ Nitrophoska™)
4 Nov. 2013	Fertiliser side dressing (36 kg-N ha^−1^ Urea)
4 Dec. 2013	Harvest
9 Dec. 2013	Incorporation residues
**Forage Sorghum** (**catch crop**)	
9 Dec. 2013	Planting
17 Jan. 2014	Incorporation residues
**Broccoli**	
10 Feb. 2014	Basal fertiliser application (42 kg-N ha^−1^ Nitrophoska™)
11 Feb. 2014	Transplanting of seedlings
14 Mar. 2014	First fertiliser side dressing (39 kg-N ha^−1^ Urea)
25 Mar. 2014	Second fertiliser side dressing (39 kg-N ha^−1^ Urea)
8, 14, 16 Apr. 2014	Harvest
30 Apr. 2014	Mulching of residues
10 Jun. 2014	Incorporation residues
**Lettuce**	
10 Jun. 2014	Basal fertiliser application (54 kg-N ha^−1^ Nitrophoska™)
11 Jun. 2014	Transplanting of seedlings
9 Jul. 2014	First fertiliser side dressing (23 kg-N ha^−1^ Urea)
1 Aug. 2014	Second fertiliser side dressing (23 kg-N ha^−1^ Urea)
26 Aug., 1 Sept. 2014	Harvest
3 Sept. 2014	Incorporation residues
